# Diffusion tensor and volumetric magnetic resonance imaging using an MR-compatible hand-induced robotic device suggests training-induced neuroplasticity in patients with chronic stroke

**DOI:** 10.3892/ijmm.2013.1476

**Published:** 2013-09-20

**Authors:** ASIMINA LAZARIDOU, LOUKAS ASTRAKAS, DIONYSSIOS MINTZOPOULOS, AZADEH KHANICHEH, ANEESH B. SINGHAL, MICHAEL A. MOSKOWITZ, BRUCE ROSEN, ARIA A. TZIKA

**Affiliations:** 1 NMR Surgical Laboratory, Department of Surgery, Massachusetts General Hospital and Shriners Burn Institute, Harvard Medical School, Boston, MA, USA; 2 Department of Radiology, Athinoula A. Martinos Center for Biomedical Imaging, Boston, MA, USA; 3 Department of Mechanical Engineering, Northeastern University, Boston, MA, USA; 4 Department of Neurology, Stroke Research Center, Massachusetts General Hospital, Harvard Medical School, Boston, MA, USA

**Keywords:** stroke, brain, robotic devices, diffusion tensor imaging, volumetric imaging, neuroplasticity

## Abstract

Stroke is the third leading cause of mortality and a frequent cause of long-term adult impairment. Improved strategies to enhance motor function in individuals with chronic disability from stroke are thus required. Post-stroke therapy may improve rehabilitation and reduce long-term disability; however, objective methods for evaluating the specific impact of rehabilitation are rare. Brain imaging studies on patients with chronic stroke have shown evidence for reorganization of areas showing functional plasticity after a stroke. In this study, we hypothesized that brain mapping using a novel magnetic resonance (MR)-compatible hand device in conjunction with state-of-the-art magnetic resonance imaging (MRI) can serve as a novel biomarker for brain plasticity induced by rehabilitative motor training in patients with chronic stroke. This hypothesis is based on the premises that robotic devices, by stimulating brain plasticity, can assist in restoring movement compromised by stroke-induced pathological changes in the brain and that these changes can then be monitored by advanced MRI. We serially examined 15 healthy controls and 4 patients with chronic stroke. We employed a combination of diffusion tensor imaging (DTI) and volumetric MRI using a 3-tesla (3T) MRI system using a 12-channel Siemens Tim coil and a novel MR-compatible hand-induced robotic device. DTI data revealed that the number of fibers and the average tract length significantly increased after 8 weeks of hand training by 110% and 64%, respectively (p<0.001). New corticospinal tract (CST) fibers projecting progressively closer to the motor cortex appeared during training. Volumetric data analysis showed a statistically significant increase in the cortical thickness of the ventral postcentral gyrus areas of patients after training relative to pre-training cortical thickness (p<0.001). We suggest that rehabilitation is possible for a longer period of time after stroke than previously thought, showing that structural plasticity is possible even after 6 months due to retained neuroplasticity. Our study is an example of personalized medicine using advanced neuroimaging methods in conjunction with robotics in the molecular medicine era.

## Introduction

Stroke affects over 780,000 individuals each year in the United States (1) and results in functional and structural brain impairment, as well as in poor motor function (2). Major efforts are underway to discover more effective methods of improving outcomes in patients with stroke in the motor and cognitive arenas (3). As a result, following rehabilitation, the majority of patients have partially recovered or are left with significant physical dysfunctions (4–6). Post-stroke rehabilitation may improve recovery and reduce long-term disability (7); however, objective methods for evaluating the specific effects of rehabilitation are required. While the findings of several studies support the hypothesis that changes in brain function accompany therapy-mediated improvements in motor skills (8–13), the spatial specificity of current evaluation methods is inadequate to allow the clear neuroanatomical localization of functional changes. In biomedical imaging research, various mechanisms have been explored based on plastic reorganization of the peri-infarct and infarct areas on axonal sprouting (14,15) and on the migration of immature neurons into the peri-infarct cortex (16). Diffusion tensor imaging (DTI)-derived measures are valid biomarkers of neuroplasticity and have been used successfully (17). Previous studies have shown that neuroplasticity may play a role in motor recovery following stroke in terms of the structural remodeling of white matter in the ipsilesional and contralesional hemispheres (18), as well as in the functional reorganization of activity in the sensorimotor cortices (19). Several studies have shown structural plasticity in stroke survivors, demonstrating the reorganization of the central nervous system, as well as experimental evidence of ‘*in vivo*’ post-stroke plasticity (20). Evidence shows that the cerebral cortex undergoes significant structural plasticity for several weeks to months following stroke (21). The reorganization taking place in the central nervous system possibly includes both cellular and anatomical phenomena, as well modifications of synaptic efficacy within neuronal networks (22). Additionally, plastic functional reorganization involves the contralesional supplementary motor area (SMA) and premotor cortex (23) and potentially the ipsilesional primary motor cortex (24). Other clinical studies have shown the benefits of using robot-assisted therapy in patients during neurological recovery (25–36). The incremental improvements in clinical scales following intensive robotic therapy, although minimal, are statistically significant and certainly meaningful to patients (32,37–39). It has been demonstrated that neurological deficits may be better predicted and more precisely characterized by incorporating brain maps of injury assessed using magnetic resonance imaging (MRI) (40) and that neurorehabilitation with a robotic devise is more beneficial than conventional paradigms (41). Brain maps can provide insight into which parts of a system are still functioning, thereby potentially providing information not evident from clinical observations (42). A recent study provided additional support for the hypothsesis that extensive time-dependent anatomical changes occur in residual tissue and must be considered when evaluating plasticity-related cortical changes associated with post-stroke recovery of function (43).

The aim of the present study was to examine the hypothesis that brain mapping using a novel magnetic resonance (MR)-compatible hand device in conjunction with state-of-the-art MRI can serve as a novel biomarker for brain plasticity induced by rehabilitative motor training in patients with chronic stroke. Thus, we explored brain plasticity after chronic stroke using volumetric and diffusion imaging developed in the molecular medicine era in conjunction with a novel MR-compatible hand-induced robotic device (MR_CHIROD). We challenge the longstanding view that neuroplasticity is not possible beyond 6 months post-stroke, which has been a critical barrier to progress in the field of rehabilitation in chronic stroke.

## Materials and methods

### Study design

We examined 15 healthy controls using DTI as part of an overall patient MR session, which included 3D high-resolution T1-weighted MRI, functional MRI (fMRI) and DTI; we also serially examined 4 patients with chronic stroke. All experiments were approved by the Institutional Review Board at Massachusetts General Hospital and performed at the Athinoula A. Martinos Center for Biomedical Imaging. The patients had first-ever left-sided ischemic subcortical middle cerebral artery (MCA) stroke ≥6 months prior to enrollment in this study, with no spasticity or joint stiffness. Patients trained at home and underwent serial MR evaluation at baseline (prior to training), during training and after 8 weeks of training. Training at home consisted of squeezing a gel exercise ball with the paretic hand at approximately 75% of maximum strength for 1 h/day, 3 days/week. For each patient, reference (100%) was own maximum force, defined as the force at which subjects could completely squeeze the MR_CHIROD [group max force, 128 ± 13 N (n=5, male)]. The appropriate hand exercise ball was selected after measuring maximum hand-grip strength using a dynamometer. MRI examinations were performed at baseline (prior to the commencement of training); 4 weeks later, halfway through the exercise period; another 4 weeks later; at the end of the training period; and again 4 weeks after completing the training period. All examinations were performed on a Siemens Tim Trio 3-Tesla (3T) MRI scanner.

### Description of MR_CHIROD

The design and testing of the first generation MR_CHIROD has been previously reported (44–47). A detailed description of the second generation MR_CHIROD used in this study has been previously published (48). Briefly, the MR_CHIROD mainly consists of 3 major subsystems: a) an electro-rheological fluid (ERF) based resistive element, b) handles and c) 2 sensors, including an optical encoder to measure patient-induced motion and a force sensor. Each subsystem includes several components of varying complexity. All components are optimally designed with strength and safety in mind for MR-compatibility and for regular and high-stress testing. The MR_CHIROD is configured to securely attach to the scanner table close to the subject who thus feels no weight.

### MRI examination protocol

All examinations were performed on a state-of-the-art 3T MRI system for increased signal-to-noise ratio (SNR). We used a 12-channel Siemens Tim coil and collected MR images and the examinations were completed in approximately 45 min. DTI images were acquired as part of an MR session for each patient, which included 3D high-resolution T1-weighted MRI, fMRI and DTI. In addition, a rapid, low resolution fully-sampled T1 magnetization prepared rapid gradient echo (MP-RAGE) or a fast spin-density weighted 3D fast low-angle shot (FLASH) gradient echo sequence was acquired (typical acquisition time, 6 sec) in order to guide the calculation of the generalized autocalibrating partially parallel acquisitions (GRAPPA) reconstruction parameters. Imaging parameters were as follows: sagittal orientation; 7° flip angle; echo time (TE) = 4.73 msec; repetition time (TR) = 2,530 msec; inversion time (TI) = 1,100 msec; 1-mm slice thickness; 352×352×192 matrix; GRAPPA factor = 3–6 to achieve the shortest acquisition time. Each volunteer performed the paradigm at 45%, 60% and 75% of their maximum grip strength and could fully squeeze the device at all levels. The percentage levels compensate for the performance confounds. Care was taken to minimize elbow flexion and/or reflexive motion and head motion (typically 0.1–0.4 mm). Typical imaging parameters for DTI were: 2×2×2 mm voxel size, 64 slices, 2 diffusion weightings (b = 0 sec/mm^2^, b = 1,000 sec/mm^2^), TR/TE = 8,600 msec/100 msec, 12 diffusion directions, 4 dummy scans, 10 T2 weighted images, 2 averages. The imaging sequence employs the twice-refocused spin-echo method for reduction of eddy current.

### Data analysis

To assess the thickness of cortical gray matter, we used the FreeSurfer automated tool (http://surfer.nmr.mgh.harvard.edu) and voxel-based morphometry (VBM) conducted using SPM8 calculated deviations of the brain volume of a patient and from 11 age- and gender-matched controls. The total acquisition time for DTI was 10 min. DTI fiber tract reconstruction was performed using the DTI Studio software package. Deterministic tractography was performed using the fuzzy art with add clustering technique (FACT) algorithm (49). All tracts were visualized and subsequently visually inspected for directionality and location. The regions of interest (balls of 3 mm diameter) were designated in the ascending fibers of the pons to visualize the corticospinal tract (CST). The purpose of this analysis was to probe alterations in diffusion-based tractography, and consequently, to demonstrate changes in structural plasticity in addition to the functional changes we observed in the brains of the chronic stroke patients as a result of hand training. We reconstructed the CST tract, selecting as seeding areas the pons, the posterior limb of the internal capsule and the motor cortex.

## Results

**Figure 1:**
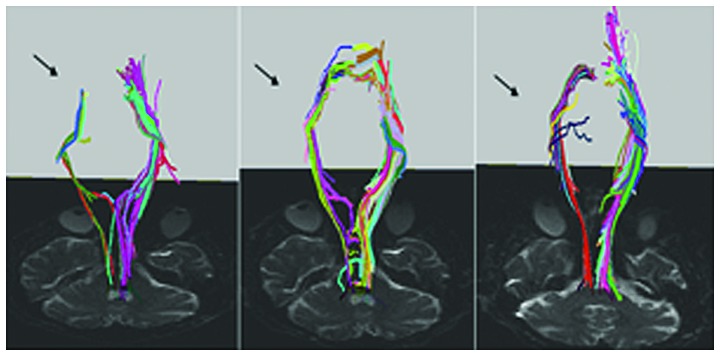
Reconstructed corticospinal tracts (CSTs) from the same stroke patient before training (left panel), after 4 weeks of training (middle panel) and after 8 weeks of training (right panel). Note that reconstructed CST fibers increased dramatically on the right side with training (arrows).

Table I summarizes the results from patients showing that the number of fibers and the average tract length were significantly altered after hand training (p<0.001). Fig. 1 depicts DTI images from a representative patient who suffered a single left-sided ischemic subcortical MCA stroke ≥6 months prior to enrollment in this study and did not have spasticity or joint stiffness. New CST fibers (arrows) projecting progressively closer to motor cortex appeared during training (Fig. 1). CST fiber (blue fibers, Fig. 1) density was altered during training and SMA recruitment was indicated from a bundle of fibers (Fig. 1).

**Figure 2:**
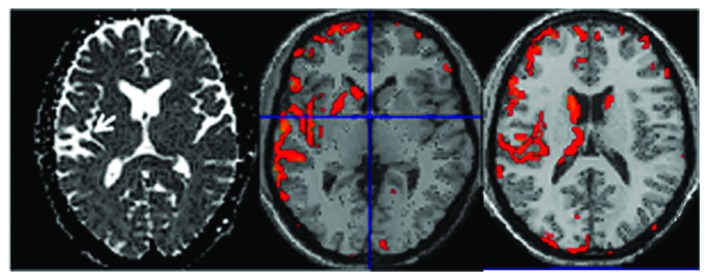
ADC maps (left image) and voxel-based morphometry (VBM) results (middle and right images) of a 55 year old patient with a left temporal stroke (white arrow). The VBM results are overlaid on a template image showing atrophy in certain areas.

**Figure 3:**
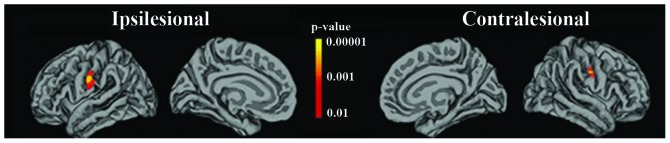
Significant regions of cortical increase induced by robotized rehabilitation training in 6 patients. Data analysis using the FreeSurfer software package showed a statistically significant increase in the cortical thickness of the ventral postcentral gyrus areas of patients after training relative to pre-training cortical thickness, thus exhibiting evidence of structural plasticity.

Our data analysis using volumetric techniques showed a decrease in cortical thickness, volume and neural density extending far beyond the stroke infarct and included most of the sensorimotor regions of the stroke and intact hemispheres (Fig. 2). We present a typical case with a stroke at the left temporal lobe showing an intense signal on the ADC map (Fig. 2, arrow). VBM was conducted using SPM8 calculated deviations of the brain volume of a patient and from 11 age- and gender-matched controls and showed cortical atrophy mainly in the affected hemisphere and noticeably even beyond the stroke region (middle and left images). Our data also showed a significant (p<0.05) increase in the cortical thickness in the ventral postcentral gyrus areas of patients after training relative to the cortical thickness before training. Our volumetric data analysis showed a significant increase in the cortical thickness of the ventral postcentral gyrus areas of patients after training relative to pre-training cortical thickness (Fig. 3).

## Discussion

In this study, we observed alterations in the number of fibers, length, density and increased cortical thickness. In addition, our volumetric data analysis showed a significant increase in the cortical thickness of the ventral postcentral gyrus areas of patients after training relative to pre-training cortical thickness. These findings suggest structural neuroplasticity in patients with chronic stroke, which may be concomitant with connectivity alterations (7–9) and are in agreement with data from previous reports of fiber tract alterations (50–52). Movement of residual tissue towards the infarct was observed, supporting the notion that extensive time-dependent morphological changes that occur in residual tissue must be considered when evaluating plasticity-related cortical changes associated with post-stroke recovery of function, which was the rationale for performing structural analysis in this study.

We were motivated to develop and use a hand device as hand movements normally play a central role in the daily lives of individuals; thus, we believe that more attention should be paid to the study of rehabilitation of hand motor function following stroke. Since a major issue in hand motor therapy is how to best restore function, interventions emphasizing intense, active and repetitive movement should be of high value. We believe that these interventions should increase strength, accuracy and functional use when applied to subjects with impairment due to stroke. For patients with chronic stroke who are in the advanced stages of recovery, rehabilitation should be aimed at returning an individual to normal activities, and should thus incorporate resistance exercises intended to support the renewed development of muscle strength. Therefore, the rationale of our approach to providing such a therapy using an MR-compatible hand robot was motivated first by the limited efforts that have been made thus far concerning robotic developments for the hand, and second by the novel combination of features that render the use of our MR-compatible hand robot promising for enhancing the effectiveness of standard post-stroke therapy.

Furthermore, the rationale for using advanced MRI methods in this study was that MRI takes advantage of anatomical, as well as functional information provided by different imaging techniques. In addition to fMRI, which depicts functional plasticity, DTI has the advantage of addressing structural brain plasticity directly by depicting alterations in the number of fibers, length and density. Thus, our rationale for using an MR-compatible hand robot in conjunction with DTI and volumetric MRI is that while robotic therapy has been shown to improve arm motor function following stroke (53,54), efforts to address brain structural plasticity have not focused on the hand (36), although, as discussed above, hand motor function is essential to everyday life. The available literature on robotic studies demonstrates clear incremental benefits in motor impairment, promoting a better outcome (36,38).

The findings of the present study suggest that intensive rehabilitation training results in neuroplasticity, which suggests that the brain is adaptable to rehabilitation even in chronic stroke. Thus, we consequently suggest that for stroke patients, rehabilitation is possible for a longer period of time following stroke than originally thought, suggesting that motor skill improvements are possible even after 6 months due to retained brain plasticity. Indeed, intensive treatment protocols for sensorimotor impairment have demonstrated benefits compared with primary care in patients with chronic stroke (26). Robotic training has been shown to enhance motor outcome in patients with stroke and the effects have been maintained for over 3 years (29). More importantly, the region of the stroke lesion may have a vital impact on the course of motor recovery in cortical and subcortical sites. Patients with mixed subcortical and cortical lesions have shown significantly greater gains in motor coordination and strength during patient rehabilitation than patients with lesions confined to the basal ganglia (55).

From a neuroscience perspective, it has been reported that stroke patients exhibit structural plasticity in the same sensorimotor cortical areas that exhibit functional plasticity (50,56,57). Our results on the cortical thickness of the ventral postcentral gyrus are in agreement with those of another study where there were regional differences in cortical thickness across the ventral postcentral gyrus areas, suggesting that the cortical thickness of the ventral postcentral gyrus areas was greater in stroke patients compared with the controls (50). Co-localized structural and functional plasticity has also been previously demonstrated in sensorimotor cortical areas of animals in response to manipulations of sensorimotor experience (58,59) involved in motor recovery after stroke (60,61). The results of a recent study on rats are consistent with our findings and the notion that extensive time-dependent anatomical changes that occur in residual tissue must be considered when evaluating plasticity-related cortical changes associated with post-stroke recovery of function (43).

We believe that our current results further extend the knowledge on brain plasticity after training and encourage further research on the specific role of structural training-induced plasticity using robotic devices (62). To this end, our findings agree with recent experimental data demonstrating changes revealed by DTI parallel histological remodeling (1,2) and recovery of function (37,63). Although it has been suggested that diffusion MR imaging may enable the assessment of brain plasticity (2,64,65), diffusion MR imaging needs to be further explored and justified for its application and diagnostic importance in humans. Other findings suggest that following stroke, brain plasticity implicates synaptogenesis, changes in function in pre-existing synapses, neurogenesis and cortical reorganization (66). The cortex, contralateral to the lesion, is active in post-stroke motor training, but the pattern of cortical activation is then normalized (67). The recovery is better if some of the relevant motor circuits are not damaged (67–69). Moreover, sensory stimulation may also enhance motor recovery (67). Of note, it has been suggested that acute and slow-growing lesions involve very different patterns of reorganization (70). Our findings support this notion in patients with chronic stroke.

Our study suggests that using advanced neuroimaging in addition to novel robotic therapies induce neuroplasticity, eventually leading to motor recovery. We believe that this approach can influence stroke practice and policy in the future. We also believe that our findings address the longstanding view that neuroplasticity was not possible beyond 6 months post-stroke which has been a critical barrier to progress in the field of neurorehabilitation in patients with chronic stroke. Moreover, the results from MR-compatible robotic devices can enhance accurate monitoring and identify biomarkers of brain plasticity that can be monitored during stroke patient rehabilitation. Therefore, our results open new horizons for the design of novel robotic devices, which would target other motor functions (i.e. arm, leg). Therefore, the current study widens the horizons for future studies focusing on verbal and memory impairments caused by stroke. Finally, our study is an example of personalized medicine using advanced neuroimaging methods in conjunction with robotics in the molecular medicine era.

## Figures and Tables

**Table I:** Comparison of CST fibers of the affected hemisphere before and after 2 months of training.

Affected fibers	Average no. ± SD	Average length ± SD (mm)
Before training (baseline)	46±8.1^a^	43.6±3.6
After training	96.8±7.1	71.4±4.5
Percentage change from baseline	110.4^b^,^c^	63.7^c^
p-value	<0.001	<0.001

a Values are means ± SE in Hz;

b values are the percentage differences before training and after training;

c statistically significant (t-test).

## References

[1] Towfighi A, Markovic D, Ovbiagele B (2012). Impact of a healthy lifestyle on all-cause and cardiovascular mortality after stroke in the USA. J Neurol Neurosurg Psychiatry.

[2] Cho HM, Choi BY, Chang CH (2012). The clinical characteristics of motor function in chronic hemiparetic stroke patients with complete corticospinal tract injury. NeuroRehabilitation.

[3] Han C, Wang Q, Meng PP, Qi MZ (2013). Effects of intensity of arm training on hemiplegic upper extremity motor recovery in stroke patients: a randomized controlled trial. Clin Rehabil.

[4] Notturno F, Sepe R, Caulo M, Uncini A, Committeri G (2013). Pseudocortical and dissociate discriminative sensory dysfunction in a thalamic stroke. Cortex.

[5] Pahlman U, Savborg M, Tarkowski E (2012). Cognitive dysfunction and physical activity after stroke: the Gothenburg cognitive stroke study in the elderly. J Stroke Cerebrovasc Dis.

[6] Carter AR, Patel KR, Astafiev SV (2012). Upstream dysfunction of somatomotor functional connectivity after corticospinal damage in stroke. Neurorehabil Neural Repair.

[7] Indredavik B, Slordahl SA, Bakke F, Rokseth R, Haheim LL (1997). Stroke unit treatment. Long-term effects. Stroke.

[8] Liepert J, Miltner WH, Bauder H (1998). Motor cortex plasticity during constraint-induced movement therapy in stroke patients. Neurosci Lett.

[9] Kopp B, Kunkel A, Muhlnickel W, Villringer K, Taub E, Flor H (1999). Plasticity in the motor system related to therapy-induced improvement of movement after stroke. Neuroreport.

[10] Liepert J, Bauder H, Wolfgang HR, Miltner WH, Taub E, Weiller C (2000). Treatment-induced cortical reorganization after stroke in humans. Stroke.

[11] Nelles G, Jentzen W, Jueptner M, Muller S, Diener HC (2001). Arm training induced brain plasticity in stroke studied with serial positron emission tomography. Neuroimage.

[12] Johansen-Berg H, Dawes H, Guy C, Smith SM, Wade DT, Matthews PM (2002). Correlation between motor improvements and altered fMRI activity after rehabilitative therapy. Brain.

[13] Schaechter JD, Kraft E, Hilliard TS (2002). Motor recovery and cortical reorganization after constraint-induced movement therapy in stroke patients: a preliminary study. Neurorehabil Neural Repair.

[14] Dijkhuizen RM, Singhal AB, Mandeville JB (2003). Correlation between brain reorganization, ischemic damage, and neurologic status after transient focal cerebral ischemia in rats: a functional magnetic resonance imaging study. J Neurosci.

[15] Mountz JM, Liu HG, Deutsch G (2003). Neuroimaging in cerebrovascular disorders: measurement of cerebral physiology after stroke and assessment of stroke recovery. Semin Nucl Med.

[16] Carmichael ST (2006). Cellular and molecular mechanisms of neural repair after stroke: making waves. Ann Neurol.

[17] Lindenberg R, Renga V, Zhu LL, Betzler F, Alsop D, Schlaug G (2010). Structural integrity of corticospinal motor fibers predicts motor impairment in chronic stroke. Neurology.

[18] Lee JS, Han MK, Kim SH, Kwon OK, Kim JH (2005). Fiber tracking by diffusion tensor imaging in corticospinal tract stroke: Topographical correlation with clinical symptoms. Neuroimage.

[19] Stinear CM, Barber PA, Smale PR, Coxon JP, Fleming MK, Byblow WD (2007). Functional potential in chronic stroke patients depends on corticospinal tract integrity. Brain.

[20] Rossini PM, Altamura C, Ferreri F (2007). Neuroimaging experimental studies on brain plasticity in recovery from stroke. Eura Medicophys.

[21] Nudo RJ (2006). Mechanisms for recovery of motor function following cortical damage. Curr Opin Neurobiol.

[22] Vaina LM, Sikoglu EM, Soloviev S (2010). Functional and anatomical profile of visual motion impairments in stroke patients correlate with fMRI in normal subjects. J Neuropsychol.

[23] Krainik A, Duffau H, Capelle L (2004). Role of the healthy hemisphere in recovery after resection of the supplementary motor area. Neurology.

[24] Duffau H, Lopes M, Sichez JP, Bitar A, Capelle L (2003). A new device for electrical stimulation mapping of the brainstem and spinal cord. Minim Invasive Neurosurg.

[25] Aisen ML, Krebs HI, Hogan N, McDowell F, Volpe BT (1997). The effect of robot-assisted therapy and rehabilitative training on motor recovery following stroke. Arch Neurol.

[26] Volpe BT, Krebs HI, Hogan N, Edelstein OL, Diels C, Aisen M (2000). A novel approach to stroke rehabilitation: robot-aided sensorimotor stimulation. Neurology.

[27] Volpe BT, Krebs HI, Hogan N, Edelsteinn L, Diels CM, Aisen ML (1999). Robot training enhanced motor outcome in patients with stroke maintained over 3 years. Neurology.

[28] Volpe BT, Krebs HI, Hogan N (2001). Is robot-aided sensorimotor training in stroke rehabilitation a realistic option?. Curr Opin Neurol.

[29] Volpe BT, Ferraro M, Lynch D (2005). Robotics and other devices in the treatment of patients recovering from stroke. Curr Neurol Neurosci Rep.

[30] Volpe BT, Ferraro M, Krebs HI, Hogan N (2002). Robotics in the rehabilitation treatment of patients with stroke. Curr Atheroscler Rep.

[31] Ferraro M, Palazzolo JJ, Krol J, Krebs HI, Hogan N, Volpe BT (2003). Robot-aided sensorimotor arm training improves outcome in patients with chronic stroke. Neurology.

[32] Fasoli SE, Krebs HI, Stein J, Frontera WR, Hughes R, Hogan N (2004). Robotic therapy for chronic motor impairments after stroke: follow-up results. Arch Phys Med Rehabil.

[33] Daly JJ, Hogan N, Perepezko EM (2005). Response to upper-limb robotics and functional neuromuscular stimulation following stroke. J Rehabil Res Dev.

[34] Macclellan LR, Bradham DD, Whitall J (2005). Robotic upper-limb neurorehabilitation in chronic stroke patients. J Rehabil Res Dev.

[35] Finley MA, Fasoli SE, Dipietro L (2005). Short-duration robotic therapy in stroke patients with severe upper-limb motor impairment. J Rehabil Res Dev.

[36] Prange GB, Jannink MJ, Groothuis-Oudshoorn CG, Hermens HJ, Ijzerman MJ (2006). Systematic review of the effect of robot-aided therapy on recovery of the hemiparetic arm after stroke. J Rehabil Res Dev.

[37] Reinkensmeyer DJ (2009). Robotic assistance for upper extremity training after stroke. Stud Health Technol Inform.

[38] Kwakkel G, Kollen BJ, Krebs HI (2008). Effects of robot-assisted therapy on upper limb recovery after stroke: a systematic review. Neurorehabil Neural Repair.

[39] Astrakas LG, Naqvi SH, Kateb B, Tzika AA (2012). Functional MRI using robotic MRI compatible devices for monitoring rehabilitation from chronic stroke in the molecular medicine era (Review). Int J Mol Med.

[40] Crafton KR, Mark AN, Cramer SC (2003). Improved understanding of cortical injury by incorporating measures of functional anatomy. Brain.

[41] Huang VS, Krakauer JW (2009). Robotic neurorehabilitation: a computational motor learning perspective. J Neuroeng Rehabil.

[42] Carey LM, Seitz RJ (2007). Functional neuroimaging in stroke recovery and neurorehabilitation: conceptual issues and perspectives. Int J Stroke.

[43] Karl JM, Alaverdashvili M, Cross AR, Whishaw IQ (2010). Thinning, movement, and volume loss of residual cortical tissue occurs after stroke in the adult rat as identified by histological and magnetic resonance imaging analysis. Neuroscience.

[44] Khanicheh A, Muto A, Triantafyllou C (2006). fMRI-compatible rehabilitation hand device. J Neuroeng Rehabl.

[45] Khanicheh A, Muto A, Triantafyllou C, Astrakas LG, Mavroidis C, Tzika A (2005). MR compatible erf-based robotic device for hand rehabilitation after stroke. Proc Intl Soc Mag Reson Med.

[46] Tzika A, Khanicheh A, Muto A, Triantafyllou C, Astrakas LG, Mavroidis C (2006). Novel rehabilitation hand robots and fMRI in Stroke [Abstract]. Eur Radiol.

[47] Khanicheh A, Mintzopoulos D, Weinberg B, Tzika AA, Mavroidis C (2007). MR_CHIROD v.2: A fMRI Compatible Mechatronic Hand Rehabilitation device.

[48] Khanicheh A, Mintzopoulos D, Weinberg B, Tzika AA, Mavroidis C (2008). MR_CHIROD v.2: magnetic resonance compatible smart hand rehabilitation device for brain imaging. IEEE Trans Neural Syst Rehabil Eng.

[49] Lazar M, Weinstein DM, Tsuruda JS (2003). White matter tractography using diffusion tensor deflection. Hum Brain Mapp.

[50] Schaechter JD, Moore CI, Connell BD, Rosen BR, Dijkhuizen RM (2006). Structural and functional plasticity in the somatosensory cortex of chronic stroke patients. Brain.

[51] Koganemaru S, Mima T, Thabit MN (2010). Recovery of upper-limb function due to enhanced use-dependent plasticity in chronic stroke patients. Brain.

[52] Cauraugh JH, Summers JJ (2005). Neural plasticity and bilateral movements: a rehabilitation approach for chronic stroke. Prog Neurobiol.

[53] Jack D, Boian R, Merians A (2001). Virtual reality-enhanced stroke rehabilitation. IEEE Trans Neural Syst Rehabil Eng.

[54] Hesse S, Schulte-Tigges G, Konrad M, Bardeleben A, Werner C (2003). Robot-assisted arm trainer for the passive and active practice of bilateral forearm and wrist movements in hemiparetic subjects. Arch Phys Med Rehabil.

[55] Shelton FN, Reding MJ (2001). Effect of lesion location on upper limb motor recovery after stroke. Stroke.

[56] Smania N, Picelli A, Gandolfi M, Fiaschi A, Tinazzi M (2008). Rehabilitation of sensorimotor integration deficits in balance impairment of patients with stroke hemiparesis: a before/after pilot study. Neurol Sci.

[57] Xerri C, Merzenich MM, Peterson BE, Jenkins W (1998). Plasticity of primary somatosensory cortex paralleling sensorimotor skill recovery from stroke in adult monkeys. J Neurophysiol.

[58] Kleim JA, Barbay S, Cooper NR (2002). Motor learning-dependent synaptogenesis is localized to functionally reorganized motor cortex. Neurobiol Learn Mem.

[59] Hickmott PW, Steen PA (2005). Large-scale changes in dendritic structure during reorganization of adult somatosensory cortex. Nat Neurosci.

[60] Carmichael ST, Archibeque I, Luke L, Nolan T, Momiy J, Li S (2005). Growth-associated gene expression after stroke: evidence for a growth-promoting region in peri-infarct cortex. Exp Neurol.

[61] Dijkhuizen RM, Ren J, Mandeville JB (2001). Functional magnetic resonance imaging of reorganization in rat brain after stroke. Proc Natl Acad Sci USA.

[62] Heller SL, Heier LA, Watts R (2005). Evidence of cerebral reorganization following perinatal stroke demonstrated with fMRI and DTI tractography. Clin Imaging.

[63] Lum P, Reinkensmeyer D, Mahoney R, Rymer WZ, Burgar C (2002). Robotic devices for movement therapy after stroke: current status and challenges to clinical acceptance. Top Stroke Rehabil.

[64] Granziera C, Schmahmann JD, Hadjikhani N (2009). Diffusion spectrum imaging shows the structural basis of functional cerebellar circuits in the human cerebellum in vivo. PLoS One.

[65] Thuen M, Olsen O, Berry M (2009). Combination of Mn(2+)-enhanced and diffusion tensor MR imaging gives complementary information about injury and regeneration in the adult rat optic nerve. J Magn Reson Imaging.

[66] Carmichael ST (2003). Plasticity of cortical projections after stroke. Neuroscientist.

[67] Dietrichs E (2007). Brain plasticity after stroke-implications for post-stroke rehabilitation. Tidsskr Nor Laegeforen.

[68] O’Dell MW, Lin CC, Harrison V (2009). Stroke rehabilitation: strategies to enhance motor recovery. Annu Rev Med.

[69] Ward NS (2005). Mechanisms underlying recovery of motor function after stroke. Postgrad Med J.

[70] Desmurget M, Bonnetblanc F, Duffau H (2007). Contrasting acute and slow-growing lesions: a new door to brain plasticity. Brain.

